# p66Shc Signaling Mediates Diabetes-Related Cognitive Decline

**DOI:** 10.1038/s41598-018-21426-6

**Published:** 2018-02-16

**Authors:** Yohei Minami, Noriyuki Sonoda, Eiichi Hayashida, Hiroaki Makimura, Makoto Ide, Noriko Ikeda, Masahiro Ohgidani, Takahiro A. Kato, Yoshihiro Seki, Yasutaka Maeda, Shigenobu Kanba, Ryoichi Takayanagi, Yoshihiro Ogawa, Toyoshi Inoguchi

**Affiliations:** 1 Department of Internal Medicine and Bioregulatory Science, Graduate School of Medical Sciences, Kyushu University, Fukuoka, Japan; 2 Innovation Center for Medical Redox Navigation, Incubation Center for Advanced Medical Science, Kyushu University, Fukuoka, Japan; 3 Department of Neuropsychiatry, Graduate School of Medical Sciences, Kyushu University, Fukuoka, Japan; 4 Department of Molecular Endocrinology and Metabolism, Graduate School of Medical and Dental Sciences, Tokyo Medical and Dental University, Tokyo, Japan; 5 CREST, Japan Agency for Medical Research and Development, Tokyo, Japan

## Abstract

Accumlating evidence have suggested that diabetes mellitus links dementia, notably of Alzheimer’s disease (AD). However, the underlying mechanism remains unclear. Several studies have shown oxidative stress (OS) to be one of the major factors in the pathogenesis of diabetic complications. Here we show OS involvement in brain damage in a diabetic animal model that is at least partially mediated through an AD-pathology-independent mechanism apart from amyloid-β accumulation. We investigated the contribution of the p66Shc signaling pathway to diabetes-related cognitive decline using *p66Shc* knockout (−/−) mice. *p66Shc* (−/−) mice have less OS in the brain and are resistant to diabetes-induced brain damage. Moreover, *p66Shc* (−/−) diabetic mice show significantly less cognitive dysfunction and decreased levels of OS and the numbers of microglia. This study postulates a p66Shc-mediated inflammatory cascade leading to OS as a causative pathogenic mechanism in diabetes-associated cognitive impairment that is at least partially mediated through an AD-pathology-independent mechanism.

## Introduction

Worldwide, as the population of the ages and life expectancy increases, the number of people with dementia is increasing. It is currently estimated the number of cases with dementia at more than 30 million and this number is expected to double by 2030 and more than triple by 2050^1^. In addition to aging, a number of reports showed that changes in life style and these related diseases could increase future dementia risk^2^. In particular, accumulating epidemiological and biological evidence suggests that diabetes mellitus increases the incidence of Alzheimer’s disease (AD)^3^. Hyperglycemia, vascular complications, insulin resistance and altered amyloid metabolism, all of these could affect cognitive function^3–6^. However, the molecular, physiological, and behavioral pathways involved in the development of dementia in diabetes remain unclear. Therefore, it is important to identify the underlying mechanism of diabetes-associated cognitive impairment for effective prevention and therapeutic intervention. Several pathological studies with autopsy samples have demonstrated that dementia subjects with diabetes have less AD-related neuropathology than subjects without diabetes^7^. Thus, diabetes seems to affect cognitive function through not only AD-dependent mechanisms but also AD-independent mechanisms apart from amyloid metabolism.

A number of *in vitro* and *in vivo* studies, including ours, have demonstrated that the production of reactive oxygen species (ROS) is increased systemically in the diabetic condition and generates a biased oxidation state known as oxidative stress (OS)^8–10^. Although low ROS levels are beneficial to cellular stress responses for the activation of several cellular signaling pathways, abnormally elevated ROS leads to damage to cells and organs, and eventually, to cell death, thereby ROS can be either beneficial or detrimental to health^11^.

Recently, one important pathway for ROS regulation has been discovered, mediated by the signaling protein p66Shc. p66Shc is encoded by the *ShcA* gene locus that is expressed as three isoforms of about 46, 52, and 66 kDa in mammals. Some studies have shown that p66Shc plays a pivotal role in regulating the intracellular redox balance *in vitro*^12^ and ablation of p66Shc results in less OS-induced tissue damage *in vivo*^13–15^.

In this study, we assessed the status of OS in the brain of type 1 and type 2 diabetic animal models. And to investigate the potential role of p66Shc, we generated streptozotocin (STZ)-induced diabetic mice carrying *p66Shc* knockout (−/−) gene. We found that the mutant mice were resistant to OS and produced less ROS in the brain. *p66Shc* (−/−) diabetic mice also showed significant amelioration of cognitive dysfunction, as well as decreased levels of OS and inflammatory markers. We found that these markers were associated with microglia-mediated neurotoxicity. This study postulates a p66Shc-mediated OS as a causative pathogenic mechanism in diabetes-associated cognitive impairment. To our knowledge, this is the first study showing a direct molecular association between OS and diabetes-related cognitive decline.

## Results

### Type 1 and Type 2 Diabetic Animal Models Develop Cognitive Impairment in an Age Dependent Manner

**Figure 1:**
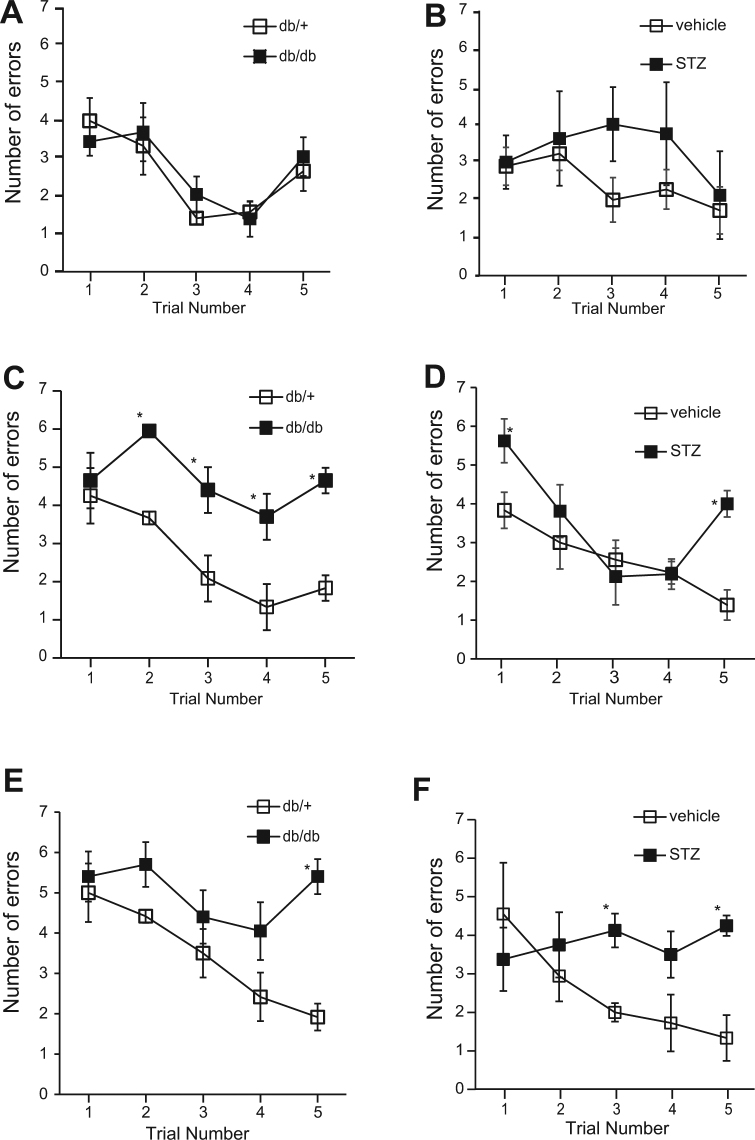
Cognitive Function is Impaired in Diabetic Mice. (**A**–**F**) The mean number of errors during radial arm water maze (RAWM) performance. (**B**,**D** and **F**) Results for male ICR mice tested 9 (vehicle, n = 9; STZ, n = 5), 14 (vehicle, n = 9; STZ, n = 8), and 22 (vehicle, n = 9; STZ, n = 4) weeks after streptozotocin (STZ) injection, respectively. (**A**,**C**,and **E**) Results for male C57BL/KsJ db/db mice aged 10, 20, and 30 weeks, respectively (db/ + , n = 6; db/db, n = 10). Four consecutive acquisition trials (trials 1–4) are followed, after 30 min, by a retention trial (trial 5). Bars represent means ± SEM. The asterisks indicate significant differences between two groups (*p < 0.05).

To generate an animal model of diabetes-impaired cognitive function for assessment of working memory and learning ability, we first examined whether cognitive impairment could develop in type 1 (STZ-induced diabetic mice) and type 2 diabetes (db/db mice) animal models using the radial arm water maze (RAWM) test. RAWM combines elements of a radial-arm maze and a Morris water maze^16^, which takes advantage of simple motivation provided by immersion into water, together with the benefits of scoring errors. In 10-week-old db/db mice, the mean number of errors was not increased compared with db/+ mice on all trials (Fig. 1A). In 20-week-old db/db mice, the mean number of errors was increased in both acquisition trials (trials 2–4) and in the retention trial (trial 5) compared with db/ + mice (Fig. 1C). In 30-week-old db/db mice, the mean number of errors was increased in the retention trial only (trial 5), compared with db/+ mice (Fig. 1E). Meanwhile, in STZ-treated mice at 9 weeks, the mean number of errors was not increased compared with vehicle treated mice (Fig. 1B). At 14 weeks, STZ-treated mice had an increased mean number of errors on one acquisition trial (trial 1) and on the retention trial (trial 5), compared with vehicle treated mice (Fig. 1D). At 22 weeks, STZ-treated mice had an increased mean number of errors on one acquisition trial (trial 3) and on the retention trial (trial 5) compared with vehicle-treated mice (Fig. 1F). These data indicate that both type 1 and type 2 diabetes models develop working memory and learning deficits in an age dependent manner that is fully consistent with previous reports^17–19^.

### Two Cognitively Impaired Diabetic Mice Models Failed to Show Accumulation of Amyloid β in Brain

**Figure 2:**
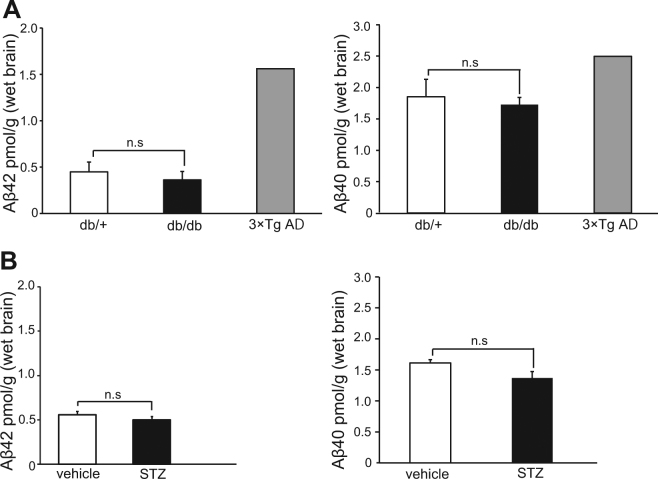
Levels of Amyloid Beta (Aβ) 42 and 40 in Brain Homogenates Are Not Increased in Diabetic Mice. Shown are the levels of Aβ42 and Aβ40 measured by ELIZA in whole brain homogenates of (**A**) 20-week-old db/db mice (db/ + , n = 8; db/db, n = 8) and of (**B**) ICR mice 22 weeks after STZ injection (vehicle, n = 8; STZ, n = 8). The triple-transgenic Alzheimer’s disease (AD) mouse model (3xTg-AD) is shown as a positive control. Bars represent means ± SEM.

Next, we evaluated whether the cognitive impairment in these diabetic mice could have been caused by amyloid β accumulation, a representative AD pathology. We measured the levels of Aβ42 and Aβ40 in whole brain homogenates using biochemical ELISA assay. These peptides are cleaved from the amyloid precursor protein (APP) by β-secretase and γ-secretase. Aβ42 in particular can form oligomeric aggregates that are thought to initiate the pathogenic cascade of AD^20^. In 20-week-old db/db mice and in 22 weeks STZ mice, both of which had already developed cognitive impairment, the levels of Aβ42 and 40 were comparable to those of age-matched, non-diabetic controls, and were less than that of a triple transgenic mouse (3XTgAD) used as a positive control (Fig. 2A and B). The 3XTgAD mouse harbors the human amyloid precursor protein (APP) Swedish mutation (APP_swe_), presenilin 1 (PS1_M146V_) and microtubule associated protein tau (Taup_301L_). Additionally, in 40-week-old db/db, the accumulation of Aβ42 and 40 were not increased compared with the control mice (data not shown). These data suggest that some mechanism other than an Aβ-dependent pathology mediates diabetes-related cognitive decline.

### Oxidative Stress and Proinflammatory Markers are Upregulated in Diabetic Mouse Brain

**Figure 3:**
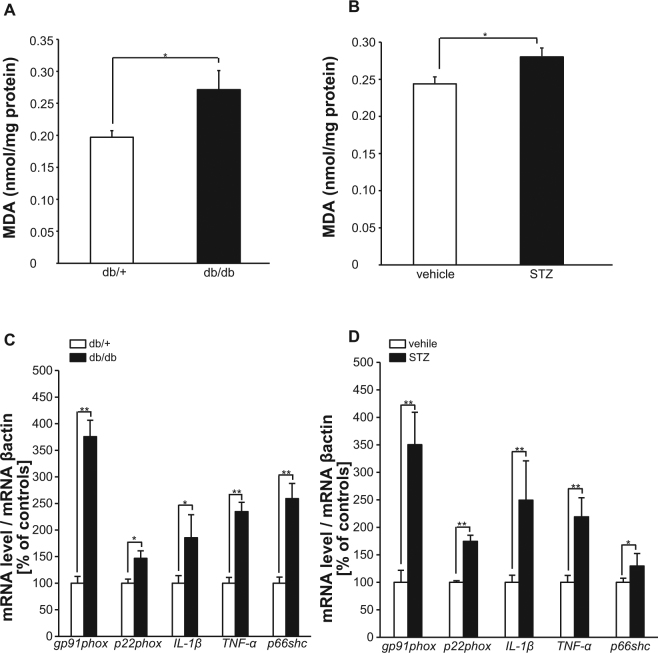
Malondialdehyde (MDA) and mRNA expressions of NAD(P)H Oxidase Components and Inflammatory Cytokines are Increased in Diabetic Mice Brain. (**A** and **B**) MDA levels measured by the thiobarbituric acid-reactive substances (TBARS) assay in whole brain homogenates, indicating oxidative stress (OS) levels. Both diabetes models produce significant OS. (A) Results for 30-week-old db/db mice (db/ + , n = 8; db/db, n = 8). (**B**) Results for ICR mice 22 weeks after STZ injection (vehicle, n = 6; STZ, n = 6). The results are expressed as nanomoles per gram of protein. The asterisks indicate significant differences relative to control mice (*p < 0.05). (**C** and **D**) Levels of mRNA expressions of NAD(P)H oxidase components (*gp91phox, p22phox*), inflammatory cytokines *(IL-1β, TNF-α*), and *p66Shc* were measured by real-time RT-PCR assay in brain homogenates from (**C**) 30-week-old db/db mice (db/ + , n = 7; db/db, n = 7) and from (**D**) ICR mice 22 weeks after STZ injection (vehicle, n = 6; STZ, n = 6). The mRNA levels are scaled to the *β-actin* level, converted to percentages of the levels in control mice, and expressed as mean ± SEM. *p < 0.05; **p < 0.01 relative to control mice.

A number of studies including ours suggest that the production of reactive oxygen species (ROS) is increased in diabetes which lead to diabetic vascular complications^8–10^. These elevated ROS in diabetes is thought to be enhanced by hyperglycemia and insulin resistance mainly through increased superoxide release from mitochondria^21^ and PKC-dependent activation of NAD(P)H oxidase^10^. Therefore, next we observed levels of malondialdehyde (MDA), a naturally occurring product of lipid peroxidation and representative indicator of OS^22^, the mRNA levels of *gp91phox*, *p22phox*, components of NAD(P)H oxidase, and *TNF-α*, *IL-1β*, inflammatory cytokines. These are significantly increased in 20 weeks aged db/db mice and 22 weeks STZ mice whole brain compared to age-matched control (Fig. 3A–D). Interestingly, *p66Shc* gene expression, known as a one of upstream positive regulator in signaling of ROS production^12,23^, was significantly increased (Fig. 3C and D). These data suggest that OS and related inflammation in brain might be associated with cognitive decline in diabetes. Especially elevation of *p66Shc*, an upstream mediator of OS prompted us to generate p66Shc knockout mice for further investigating causal role of p66Shc signaling-mediated OS in diabetes-related cognitive decline.

### Generation of *p66Shc* (−/−) Mice and their General Characteristics

**Figure 4:**
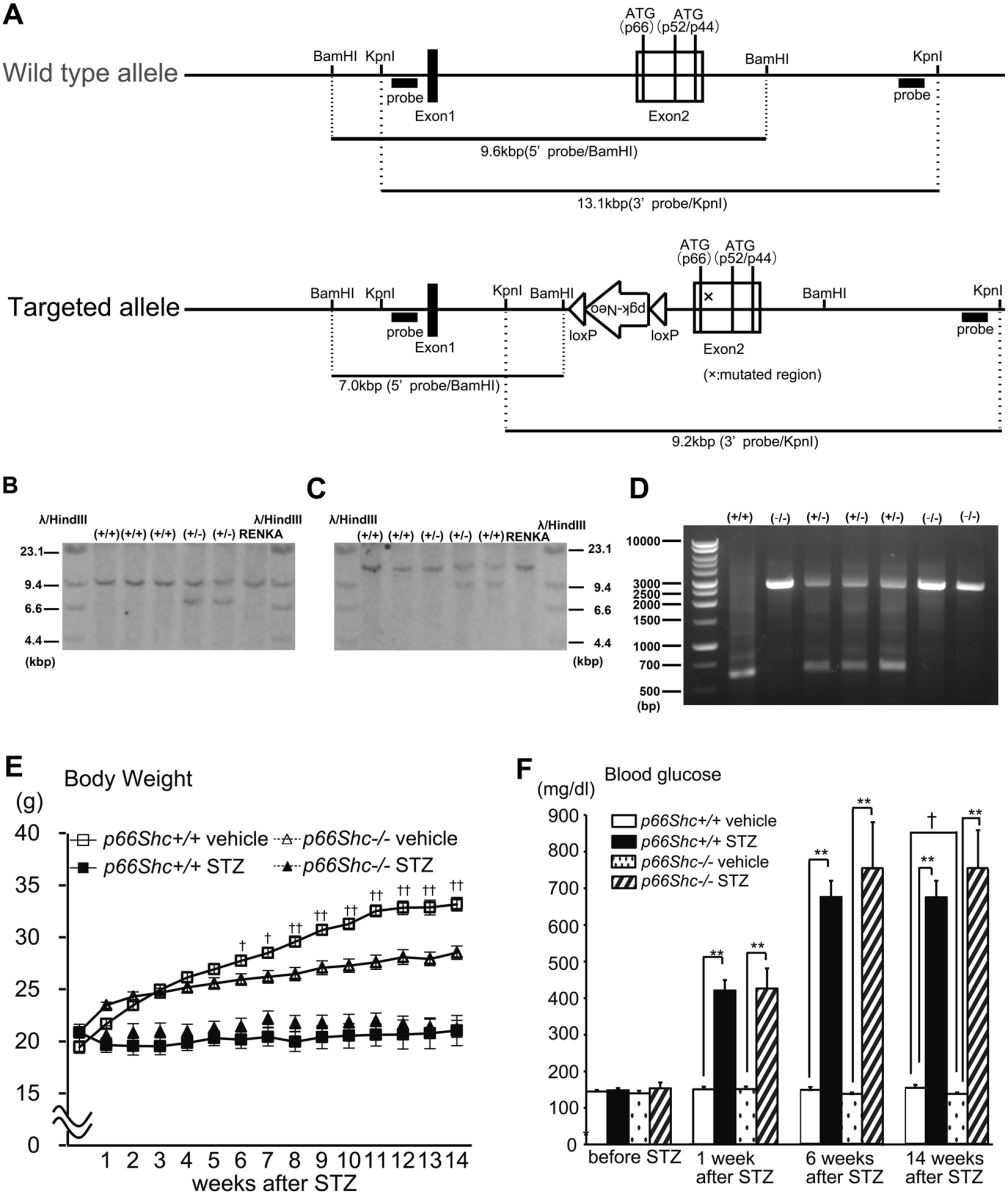
Generation of p66Shc Knockout Mice and Their Phenotypic Characterization after STZ-induction of Diabetes. (**A**–**C**) Gene-disrupted mice with targeted disruption of *p66Shc*. (**A**) Schematic presentation of the genomic structure of p66Shc knockout mice. (**B** and **C**) Southern blot analysis of embryonic stem (ES) cell clones. (+/+) Lanes are wild-type ES cell clones and (+/−) lanes are mutated ES cell clones. RENKA is the wild-type ES genome, used as the (+/+) control. (**B**) 5′ Southern blot; the targeted allele generates a 7.0 kbp BamHI fragment in contrast to the 9.6 kbp wild-type fragment. (**C**) 3′ Southern blot; the targeted allele generates a 9.2 kbp kpnI fragment in contrast to the 13.1 kbp wild-type fragment. (**D**) PCR analysis for *p66Shc* genomic DNA knockout, using the tails. Wild type, (+/+); heterozygous, (+/−); homozygous, (−/−) for *p66Shc*. A 2566 bp mutant fragment and a 616 bp wild-type fragment are detected. (**E**,**F**) Shown are body weight (g) and blood glucose concentrations (mg/dL) in p66Shc knockout mice in STZ-induced diabetes [*p66Sh*c (+/+) vehicle, n = 19; *p66Sh*c (+/+) STZ, n = 16; *p66Sh*c (−/−) vehicle, n = 10; *p66Sh*c (−/−) STZ, n = 8], Data are means ± SEM. Note that there is no significant difference in body weight comparing *p66Shc* (+/+) STZ vs. *p66Shc* (−/−) STZ, but a significant difference (p < 0.05) comparing STZ vs. vehicle in each of *p66Sh*c (+/+) and *p66Shc* (−/−) after STZ injection. (F) Note that there is no significant difference in blood glucose comparing *p66Shc* (+/+) STZ vs. *p66Shc* (−/−) STZ. ^†^p < 0.05, *p66Shc* (+/+) vehicle vs. *p66Shc* (−/−) vehicle; *p < 0.05 and **p < 0.01, STZ vs. vehicle in the same genotype.

Therefore, we generated *p66Shc* (−/−) mice and examined their working memory, learning ability, and related mechanisms, both in the presence or absence of STZ-induced diabetes. Figure 4A show our strategy for designing the targeting vectors for *p66Shc* gene deletion. An exon2 containing an initiating ATG codon was point mutated, and a reverse-oriented Neo-cassette was inserted. *p66Shc* targeting was confirmed in embryonic stem (ES) cells by Southern blot analysis (Fig. 4B and C). Generation of *p66Shc* (−/−) mice was confirmed by PCR analysis from genomic tail DNA (Fig. 4D). And deleted protein expression of p66Shc was confirmed by western blotting. One of the most abundant expression of p66Shc proteins is found in liver in wild type. We examined *p66Shc* (−/−) mice using liver homogenates to confirm deleted protein expression (Fig. S1). Next, we induced diabetes in *p66Shc* (−/−) mice and age-matched *p66Shc* (+/+; wild type) mice by i.p. injection of STZ, or of vehicle to provide further controls. The body weight (BW) of STZ-treated mice was significantly decreased and the blood glucose level significantly increased compared with those of vehicle-treated mice in both *p66Shc* (+/+) and (−/−) mice (Fig. 4E and F). We observed the BW and blood glucose of *p66shc* (−/−) vehicle treated mice are significantly decreased compared to *p66shc* (+/+) vehicle treated mice (Fig. 4E and F), since *p66shc* (−/−) mice have increased metabolic rate, decreased fat mass and decreased insulin resistance^12,24^. However, we found no significant differences in BW or blood glucose levels between STZ-treated *p66Shc* (+/+) and *p66Shc* (−/−) mice (Fig. 4E).

### Cognitive Impairment is Ameliorated and Oxidative Stress is Decreased in *p66Shc* (−/−), STZ-induced Diabetic Mouse Brain

**Figure 5:**
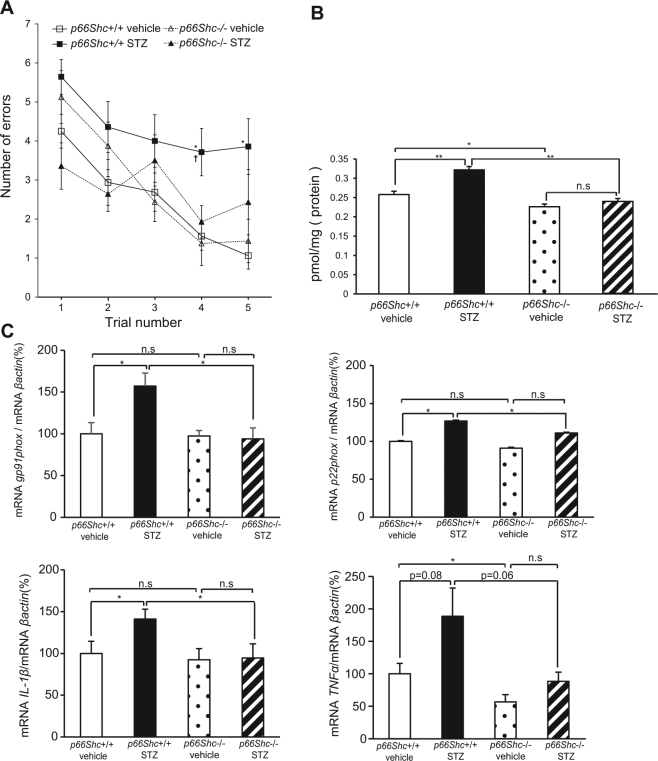
Cognitive Impairment on RAWM, Elevation of MDA, and Elevation of mRNA Expressions of NAD(P)H Oxidase Components and Inflammatory Cytokines are Ameliorated in Diabetic Mice by *p66Shc* Gene Knockout. (**A**) Mean numbers of errors during RAWM performance in p66Shc knockout mice 14 weeks after STZ injection [*p66Sh*c (+/+) vehicle, n = 8; *p66Sh*c (+/+) STZ, n = 7; *p66Sh*c (−/−) vehicle, n = 8; *p66Sh*c (−/−) STZ, n = 7]. (B) MDA levels measured by the TBARS assay in whole brain homogenates from p66Shc knockout mice, 22 weeks after STZ injection [*p66Sh*c (+/+) vehicle, n = 8; *p66Sh*c (+/+) STZ, n = 8; *p66Sh*c (−/−) vehicle, n = 8; *p66Sh*c (−/−) STZ, n = 8]. (**C**) Levels of mRNA expressions of NAD(P)H oxidase components (*gp91phox, p22phox*) and inflammatory cytokines *(IL-1β, TNF-α*) measured by real-time RT-PCR assay in whole brain homogenates from p66Shc knockout mice, 14 weeks after STZ injection [*p66Sh*c (+/+) vehicle, n = 8; *p66Sh*c (+/+) STZ, n = 8; *p66Sh*c (−/−) vehicle, n = 8; *p66Sh*c (−/−) STZ, n = 7]. *p < 0.05; **p < 0.01.

We performed RAWM testing to evaluate cognitive function in *p66Shc* (−/−) and (+/+) mice with and without diabetes. In *p66Shc* (+/+), STZ-treated mice, the mean number of errors (trials 4 and 5) was significantly increased compared with *p66Shc* (+/+), vehicle-treated mice (Fig. 5A), which is consistent with pilot study data shown in Fig. 1. Interestingly, we observed significantly decreased (p < 0.05) errors in *p66Shc* (−/−), STZ-treated (diabetic) mice compared with *p66Shc* (+/+), STZ-treated mice. Next, we measured the levels of OS and proinflammatory markers. In parallel with the amelioration of the cognitive impairment, diabetic brain MDA levels and the mRNA levels of *gp91phox*, *p22phox*, and *IL-1β* were significantly decreased, to non-diabetic control levels (Fig. 5B and C). The level of *TNF-α* was increased with diabetes, but was not statistically significant in this experiment (p = 0.06).

### p66Shc Protein is Mainly Expressed in Microglia in the Brain

**Figure 6:**
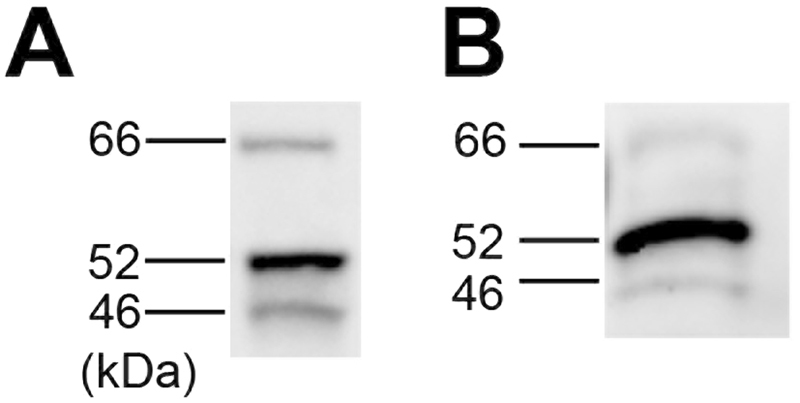
p66Shc Protein is Highly Expressed in Microglial Cells in the Brain. Western blotting results using anti-Shc protein antibody, (**A**) with total protein extracted from microglial HAPI (highly aggressively proliferating immortalized) cells, and (**B**) with total protein extracted from brain microglia of C57B/6 mice. The full-length blotting images are presented in Fig. S2.

To investigate the mechanism restoring cognitive function in diabetic mice with p66Shc deletion, we next examined what type of cells express p66Shc in the brain. As there is no specific antibody to p66Shc available for immunohistochemistry, we examined the expression of p66Shc in the brain by western blotting. p66Shc protein expression could not be detected in the whole brain tissue of *p66Shc* (+/+) mice (data not shown). This is consistent with a previous report showing that p66Shc protein is little expressed in brain tissue in mice^25^. Since a large proportion of the brain is occupied by neurons, we expected that the p66Shc protein might be expressed in some other cell type such as glia. Using an extraction technique recently developed by our group^26^, we extracted and purified microglial cells from *p66Shc* (+/+) mouse brain. Western blotting showed the p66Shc protein to be well expressed in these cells, as well as in an authentic microglial cell line called HAPI cells (highly aggressively proliferating immortalized) (Fig. 6A and B).

### Effect of p66Shc Knockout on Microglia in the Brains of db/db and STZ-treated Diabetic Mice

**Figure 7:**
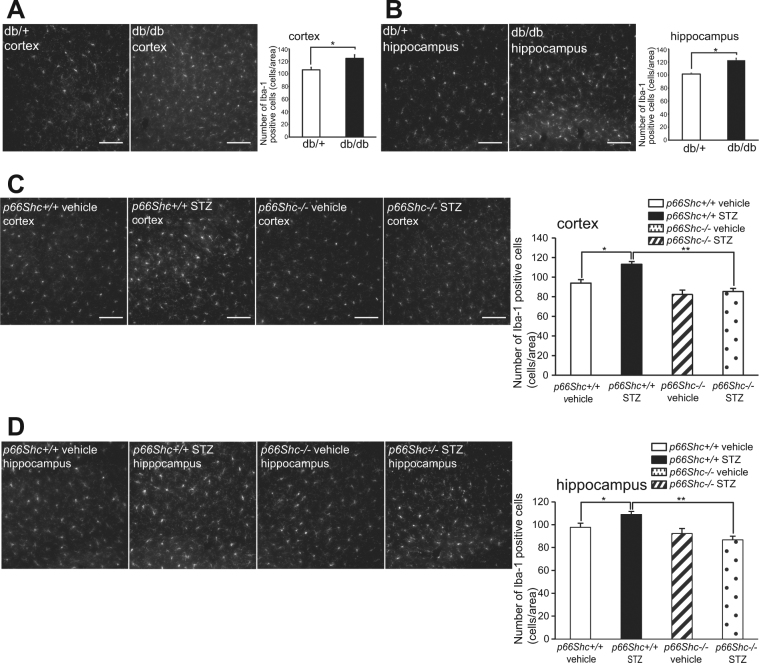
Brain Microglia are Proliferated in Diabetic Mice and Suppressed by *p66Shc* Gene Knockout. (**A** and **B**) Counts of Iba1–positive cells in 30-week-old db/db mice (cortex, db/ + , n = 4; cortex, db/db, n = 4; hippocampus, db/+ , n = 6; hippocampus, db/db, n = 6). (**C** and **D**) Counts of Iba1–positive cells in p66Shc knockout mice 14 weeks after STZ injection [*p66Sh*c (+/+) vehicle, n = 6; *p66Sh*c (+/+) STZ, n = 6; *p66Sh*c (−/−) vehicle, n = 6; *p66Sh*c (−/−) STZ, n = 6]. Bars represent means ± SEM. *p < 0.05; **p < 0.01.

Microglia, immune cells in the brain, continuously monitor the brain environment^27,28^. In response to certain cues such as brain injury or immunological challenges, microglia are readily activated and triggered into proliferation^29^. We therefore counted the number of microglial cells after staining with an antibody to ionized calcium binding adaptor molecule 1 (Iba1). Iba1 is a protein restricted to microglia/macrophages^30^, and is up-regulated in activated microglia^31^. In db/db mice, the number of Iba1-positive microglial cells was significantly increased in both hippocampus and cortex compared with that of db/ + mice (Fig. 7A and B). Similarly, the number of Iba1-positive microglial cells in *p66Shc* (+/+), STZ-treated mice was significantly increased compared with *p66Shc* (+/+), vehicle-treated mice 14 weeks after STZ injection. Interestingly, the number of Iba1-positive microglial cells in *p66Shc* (−/−), STZ-treated mice was markedly decreased compared with *p66Shc* (+/+), STZ-treated mice (Fig. 7C and D).

## Discussion

In this study, we found that middle-aged to elderly diabetic mice showed an elevation of oxidative stress (OS) in the brain without amyloid-β accumulation before onset of cognitive impairment. Furthermore, p66Shc knockout mice, which are resistant to OS, were protected from diabetes-induced cognitive decline in correlation with decreased OS and proinflammatory markers. These findings support a causative role of p66Shc-mediated OS in diabetes-related dementia. Notably, we observed that changes in microglial number in STZ-induced diabetes were normalized in p66Shc knockout mice, suggesting an important role for this type of cell in disease development. This study provides evidence for a role of p66Shc-mediated OS in diabetes related-dementia and this is at least partially independent of the known amyloid-β–mediated neurodegenerative pathway of Alzheimer’s disease (AD).

Recent epidemiological studies demonstrate that diabetes is an important risk factor for dementia^3^. In addition, several animal studies with both type 1 and type 2 diabetic models show progressive cognitive declines in an age-dependent manner^17–19^. Consistent with previous findings, both type 1 (STZ-induced diabetic mouse) and type 2 (db/db mouse) models showed cognitive decline dependent on age in this study (Fig. 1). However, at the time point when memory impairment was demonstrated by a spatial memory test, Aβ40 and Aβ42, the major forms of amyloid, were not elevated, suggesting an alternative pathway for the development cognitive decline in diabetes. Consistent with our findings, several human pathological studies with autopsy samples suggest that dementia in diabetic patients is due not only to AD pathology-dependent mechanisms but also to AD-independent mechanisms^7^. A recent clinical brain study with MRI, SPECT, and amyloid PET imaging support this notion^32,33^. These observations taken together with ours strongly suggest that the underlying mechanism of diabetes-related dementia is at least partially different from that of typical AD; diabetes may promote dementia by unique pathways independent of AD pathology.

High levels of ROS cause damage to cells by modifying DNA, proteins, or lipids and can lead to arrest of proliferation and eventually apoptosis. The brain is thought to be especially sensitive to OS-induced damage due to its high levels of polyunsaturated fatty acids, high oxygen consumption, and poor antioxidant defenses. In this study, we observed a prominent elevation of OS markers in both type 1 and type 2, middle-aged diabetic models. Notably, the expression levels of p66Shc, an adaptor/signaling protein mediating ROS production, were increased in diabetic mice compared with non-diabetic controls. These observations prompted us to investigate brain function in p66Shc knockout mice with STZ-induced diabetes. Interestingly, p66Shc (−/−), diabetic mice were highly protected from cognitive decline, suggesting that p66Shc-mediated OS and related inflammation is a major mechanism of diabetes-related dementia. Pelicci and co-workers first showed a p66Shc adaptor protein that controls oxidative stress response^12^. Afterwards, several lines of evidence, including knockout mouse studies, demonstrated that p66Shc is an upstream regulator in OS-induced tissue damage.

Considering the mechanism of the p66Shc-mediated brain damage pathway, we have highlighted microglial activity. We found that p66Shc protein expression is low on whole-brain basis, but relatively abundant in microglial cells (Fig. 6A and B). It is well known that the pro-inflammatory activity of microglia correlates with their proliferative activity^29^. We observed that the number of Iba1-positive (i.e., microglial) cells was increased in STZ-induced mice and in db/db mouse brain (Fig. 7A and B) raising the possibility that the activity of microglia may be related to cognitive decline in diabetic mice and that these changes were normalized in STZ-induced diabetic mice carrying the *p66Shc* (−/−) genotype. We have recently shown that microglial activation induce cognitive decline via hippocampal microglial activation by even a short period of stress in mice^26^. To our knowledge, the role of p66Shc in microglia is yet unknown. Microglia have common features with macrophages. We have previously shown that microglia release superoxide radicals via NOX2^34^. NOX enzymes are not only restricted to microglia but also expressed in neurons, astrocytes, and the neurovascular system^35^. Several studies have also demonstrated that NOX2 expression in microglia is markedly increased at an activated state in various neurodegenerative diseases^36,37^. Interestingly, production of superoxide by NAD(P)H oxidase is decreased in macrophages from long-lived p66Shc knockout mice^38^. Microglial activities represent critical lines of defense against the development of neurodegenerative disease; microglia clear misfolded proteins, elaborate trophic and regenerative factors, and regulate and terminate toxic inflammation^39,40^. Therefore, we speculate that the ameliorated diabetes-related cognitive decline in p66Shc knockout mice is attributable to a reduction of p66Shc-mediated ROS production by microglia.

Another possibility is that the relative cognitive improvement seen in STZ-induced *p66Shc* (−/−) mice was due to a reduction in systematic oxidative damage and inflammation. It is consistent with findings that p66Shc knockout mice also have reduced systemic OS^12^ and are resistant to atherosclerosis^41^, oxidant-related endothelial dysfunction^13,42^, and high-fat diet-induced obesity^24,41^. Consistent with previous reports, we here observe lower body weights and less insulin resistance in p66Shc knockout mice compared with wild type (Fig. 4E and F). In this study, we found that p66Shc signaling contributes to cognitive decline in high blood glucose model (STZ-induced diabetic mice) and further investigation is required for whether this is applicable to all type of diabetes such as high fat-induced insulin resistant state.

Here we identified a novel p66Shc-mediated pathway, which involves elevating OS in the brain, leading to cognitive impairment in diabetic animal models. And that clinical regulation of redox state by manipulating p66Shc signaling may open up new approaches for preventing and intervening diabetes-associated dementia.

## Methods

### Animals

All mice were purchased from Charles River (Yokohama, Japan), and bred under pathogen-free conditions at the Kyushu University Animal Center (Fukuoka, Japan). All protocols were approved by the Ethics Committee of Kyushu University Graduate School of Medicine. All methods were performed in accordance with the approved institutional guidelines. Every effort was made to minimize the number of animals used and their suffering.

Male C57BL/KsJ, db/db mice were used for the type 2 diabetes model. Their age-matched, lean littermates of db/ + genotype were used as controls. For the type 1 diabetes model, male ICR mice were made diabetic by administering a single injection of streptozotocin (STZ; Sigma-Aldrich, St. Louis, MO) intraperitoneally at a dose of 100 mg/kg in 0.1 M citrate buffer (CIT), pH 4.5. Mice given injections of CIT alone served as the vehicle controls. Diabetes was confirmed by a finding of hyperglycemia (>250 mg/dL blood glucose). Body weight and blood glucose concentrations in type 1 and type 2 diabetic mice are given in Supplemental Materials (Table S2). Gene-disrupted mice with a targeted disruption of *p66Shc* were custom-generated by TransGenic, Inc. (Kumamoto, Japan). See also Fig. 4. Male *p66Shc* (−/−) mice and age-matched *p66Shc* (+/+) mice were made diabetic by administering 80 mg/kg of STZ or CIT alone intraperitoneally for three consecutive days.

### Radial Arm Water Maze

Learning and memory were assessed using the RAWM. The protocol and apparatus for this test have been described previously^43^. On each trial, the mouse was started in one arm and allowed to swim for up to 1 min until it reached the platform. The number of errors made before the mouse reached the platform was recorded. The mouse was allowed to stay on the platform for 30 s between trials. After the fourth trial, the mouse was placed in a cage for 30 min, and then returned to the maze and started on the fifth trial, which assessed memory retention. After three consecutive days of training, the error score was determined as the score on the fifth trial averaged over the next 2 days of testing.

### Aβ ELISA

The brain levels of Aβ42 and Aβ40 were determined in homogenates of whole mouse brains by ELISA (Wako, Osaka, Japan) according to the manufacturer’s instructions. The 3XTgAD mouse used as a positive control was kindly provided by Dr Yasumasa Ohyagi from the department neurology, Kyushu University, Japan.

### Brain Lipid Peroxidation

The brain levels of lipid peroxidation were estimated in whole mouse brain homogenates as malondialdehyde (MDA) concentration using the Thiobarbituric acid reactive substances (TBARS) assay kit (Cayman Chem, Ann Arbor, MI, USA) according to the manufacturer’s instructions.

### RNA Extraction and Quantitative RT-PCR

Total RNA was isolated from whole brain using the SV Total RNA Isolation System (Promega, Madison, WI, USA) following the manufacturer’s instructions. The mRNA levels were measured by quantitative RT-PCR using the GoTaq Master Mix (Promega). mRNA levels were also quantified by real-time RT-PCR using a Roche Light Cycler 480 iCycler system. The mRNA expression levels of each gene were normalized to the expression level of the housekeeping gene *β-actin*. The specific primer sequences used for target and housekeeping genes are given in Supplemental Materials (Table S1).

### Western Blotting Analysis

To prepare total protein extracts for western blotting analysis of p66Shc, highly aggressively proliferating immortalized (HAPI) cells were lysed in lysis buffer (50 mM Tris-HCl, pH 7.4, 150 mM NaCl, 5 mM NaF, 0.25 mM EDTA, pH 8.0, 1% deoxycholic acid, 1% Triton X-100, 1 mM sodium orthovanadate) supplemented with a protease inhibitor mixture and a phosphatase inhibitor mixture (both from Sigma). Samples were separated on discontinuous 4%–15% sodium dodecyl sulfate-polyacrylamide gels and transferred to polyvinyl difluoride membranes (Bio-Rad). The brain primary microglial cells from C57BL/6 mice were isolated according to a protocol described previously^26^, and were homogenized in lysis buffer (0.25 M sucrose, 1 mM EDTA) supplemented with a protease inhibitor cocktail and phosphatase inhibitors (both from Sigma, St Louis, MO, USA), then centrifuged for 10 min at 16,000 rpm. Protein concentrations were determined using a Quick Start^TM^ Bradford Protein Assay (Bio-Rad Hercules, CA, USA). Then 20 μg of protein per lane were separated on discontinuous 4%–15% sodium dodecyl sulfate-polyacrylamide gels and transferred to polyvinyl difluoride membranes (Bio-Rad). After blocking nonspecific binding, the membranes were incubated overnight at 4 °C with anti-Shc (1:1000; BD Transduction Laboratories) antibody, followed by horseradish peroxidase-conjugated sheep anti-mouse IgG (1:10,000; Amersham Pharmacia Biosciences, Buckinghamshire, UK) or donkey anti-rabbit IgG (1:10,000; Amersham) as secondary antibodies. We used the ECL Plus system (Amersham) for protein detection.

### Tissue Processing

Animals were anesthetized with isoflurane (4% for induction, 1-2% for maintenance) mixed with medical air (750 mL/min) and blown into a nose cone fitted to the animal’s head. They were then perfused transcardially with phosphate buffered saline (PBS, pH 7.4) followed by fixative: a mixture of 4% paraformaldehyde (PFA) and 0.1% glutaraldehyde in 0.1 M phosphate buffer (PB) for immunostaining. The brains were left *in situ* for 3 h at room temperature before being removed from the skull. The brains were post fixed by immersion in 4% PFA overnight at 4 °C, then in 20% sucrose (pH 7.4) for 24 h at 4 °C. The brains were then cut into 50-μm-thick sections on a vibrating microtome (CM1950; Leica Microsystems, Wetzlar, Germany). To avoid deformation, sections were processed free-floating with extreme caution.

### Immunofluorescence Procedure

The brain sections were incubated with 1.0% bovine serum albumin in PBS containing 0.3% Triton-X 100 and 0.05% sodium azide for 30 min at room temperature. Then they were incubated for 3 days at room temperature with rabbit polyclonal anti-Iba1 (ionized calcium binding adaptor protein 1) antibody (1:10,000; Wako, Pure Chemical Industries, Osaka, Japan). They were then incubated with fluorescein isothiocyanate (FITC)-conjugated donkey anti-goat IgG antibody (1:300; Jackson ImmunoResearch Laboratories) for 12 h at 4 °C in a dark chamber. The sections were then counterstained with Hoechst 33258 (Invitrogen, Carlsbad, CA, USA) in PBS for 15 min in a dark chamber. After washing with PBS, the sections were mounted in Vectashield (Vector laboratories, Peterborough, UK) and examined as described below.

### Cell Counting and Morphological Analysis of Iba1-positive Cells

Twenty pieces of a Z-stack image were acquired from 40-μm-thick sections separated by 2-μm intervals, and converted to one z-projection image. The images for counting Iba1-positive cells were examined using a fluorescence microscope (model BZ-9000, Keyence, Osaka, Japan). We counted the Hoechst 33258-stained nuclei of Iba1-positive microglia using the Cell Counter plugin of ImageJ 1.44 (NIMH; Bethesda, MD, USA). The reported cell number is the average of cell numbers from four images. The Z-projection image acquired from Z-stack images was also analyzed using ImageJ 1.44.

### Statistical Analysis

Comparisons between data sets with two groups were evaluated using an unpaired Student’s *t*-test. p-Values of less than or equal to 0.05 were considered to indicate statistical significance. Results are presented as mean ± SEM. ∗p < 0.05; ∗∗p < 0.01; ^†^p < 0.05; ^††^p < 0.01. Asterisks indicate comparison of treatments, whereas daggers indicate comparison of genotypes.

## Electronic supplementary material

Supplementary Informaton
