# Supplementary material for: p66Shc Signaling Mediates Diabetes-Related Cognitive Decline
Source: Sci Rep. 2018 Feb 16;8:3213. doi: 10.1038/s41598-018-21426-6 (PMC5816624; doi:10.1038/s41598-018-21426-6)
Supplement: Supplementary file 1 — Supplementary Informaton [file 41598_2018_21426_MOESM1_ESM.pdf]

Supplementary Information

p66Shc Signaling Mediates Diabetes-Related Cognitive Decline

Yohei Minami[1], Noriyuki Sonoda[1,2,6], Eiichi Hayashida[1], Hiroaki Makimura[1], Makoto Ide[1], Noriko Ikeda[1], Masahiro Ohgidani[3], Takahiro A. Kato[2,3], Yoshihiro Seki[3], Yasutaka Maeda[1,2], Shigenobu Kanba[3], Ryoichi Takayanagi[1], Yoshihiro Ogawa[1,4,5], Toyoshi Inoguchi[1,2]

[1]Department of Internal Medicine and Bioregulatory Science, Graduate School of Medical Sciences, Kyushu University, Fukuoka, Japan, [2]Innovation Center for Medical Redox Navigation, Kyushu University, Fukuoka, Japan, [3]Department of Neuropsychiatry, Graduate School of Medical Sciences, Kyushu University, Fukuoka, Japan, [4]Department of Molecular Endocrinology and Metabolism, Graduate School of Medical and Dental Sciences, Tokyo Medical and Dental University, Tokyo, Japan, [5]CREST, Japan Agency for Medical Research and Development, Tokyo, Japan [6]Lead Contact.

Correspondence and reprint requests should be addressed to Noriyuki Sonoda. (E. mail: noriyuki@intmed3.med.kyushu-u.ac.jp)

Table S1: Nucleotide Sequences of Primers. Related to Figure 3, 5

Gene	Forward primer (5’→3’)	Reverse primer (5’→3’)
gp91phox	ACTGCGGAGAGTTTGGAAGA	GGTGATGACCACCTTTTGCT
p22phox	TGGCTACTGCTGGACGTTTCAC	CTCCAGCAGACAGATGAGCACAC
IL-1β	AAATACCTGTGGCCTTGGGC	CTTGGGATCCACACTCTCCAG
TNF-α	CTCCTGGCCAACGGCATGGAT	ATCGGCTGACGGTGTGGGTG
p66Shc	CCCCAAGCCGAAGTACAACCCA	TCCGGGGAAAGAAGGAACACAGG
β-Actin	TGACAGGATGCAGAAGGAGA	GCTGGAAGGTGGACAGTGAG

Table S2: Body Weight and Blood Glucose in Type 1 and Type 2 diabetic mice. Related to Figure 1 to 3.

	age (weeks)	body weight (g)	Blood glucose (mg/dL)
Type 1 diabetic mice (ICR)			
diabetic (STZ-induced), n = 9	7weeks aged	33.6±0.3	133.0±5.6
control (vehicle), n = 9	(before STZ)	33.8±0.5	137.8±8.3
diabetic (STZ-induced), n = 9	21weeks aged	41.4±1.3*	681.2±27.9*
control (vehicle), n = 9	(14 weeks after STZ)	48.0±0.6	164.3±3.8
Type 2 diabetic mice (db/db)			
diabetic (db/db), n = 10	10 weeks aged	44.9±0.5*	543.9±14.7*
control (db/+), n = 6		27.6±0.2	128.7±13.6
diabetic (db/db), n = 10	30weeks aged	57.0±2.4*	487.4±128.0*
control (db/+), n = 6		32.3±0.3	116.5±8.8

Body weight and blood glucose concentrations in type 1 and type 2 diabetic mice. Data are means ± SE. *P < 0.05 vs. control.

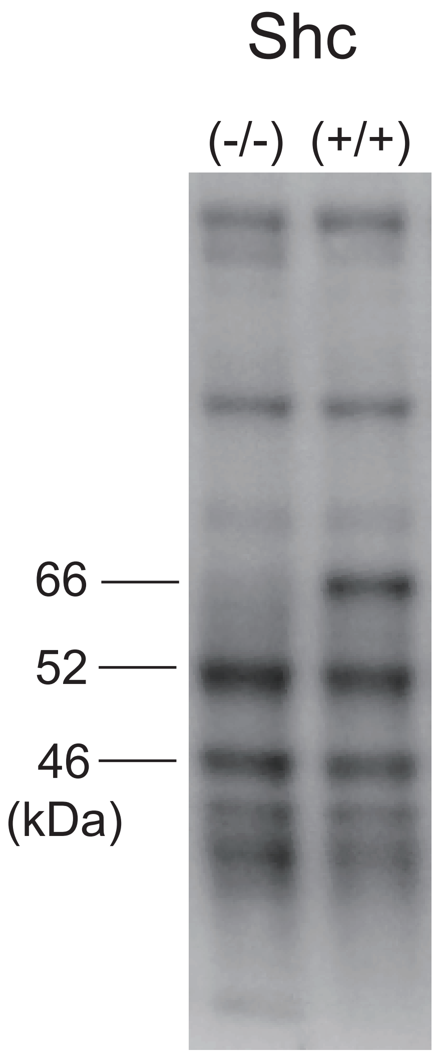

Figure S1: Western blot analysis to confirm deleted protein expression of p66Shc. Related to Figure 4.

Western blotting results using anti-Shc protein antibody, with total protein extracted from liver homogenates of *p66Shc (-/-)* and *p66shc (+/+)* mice.

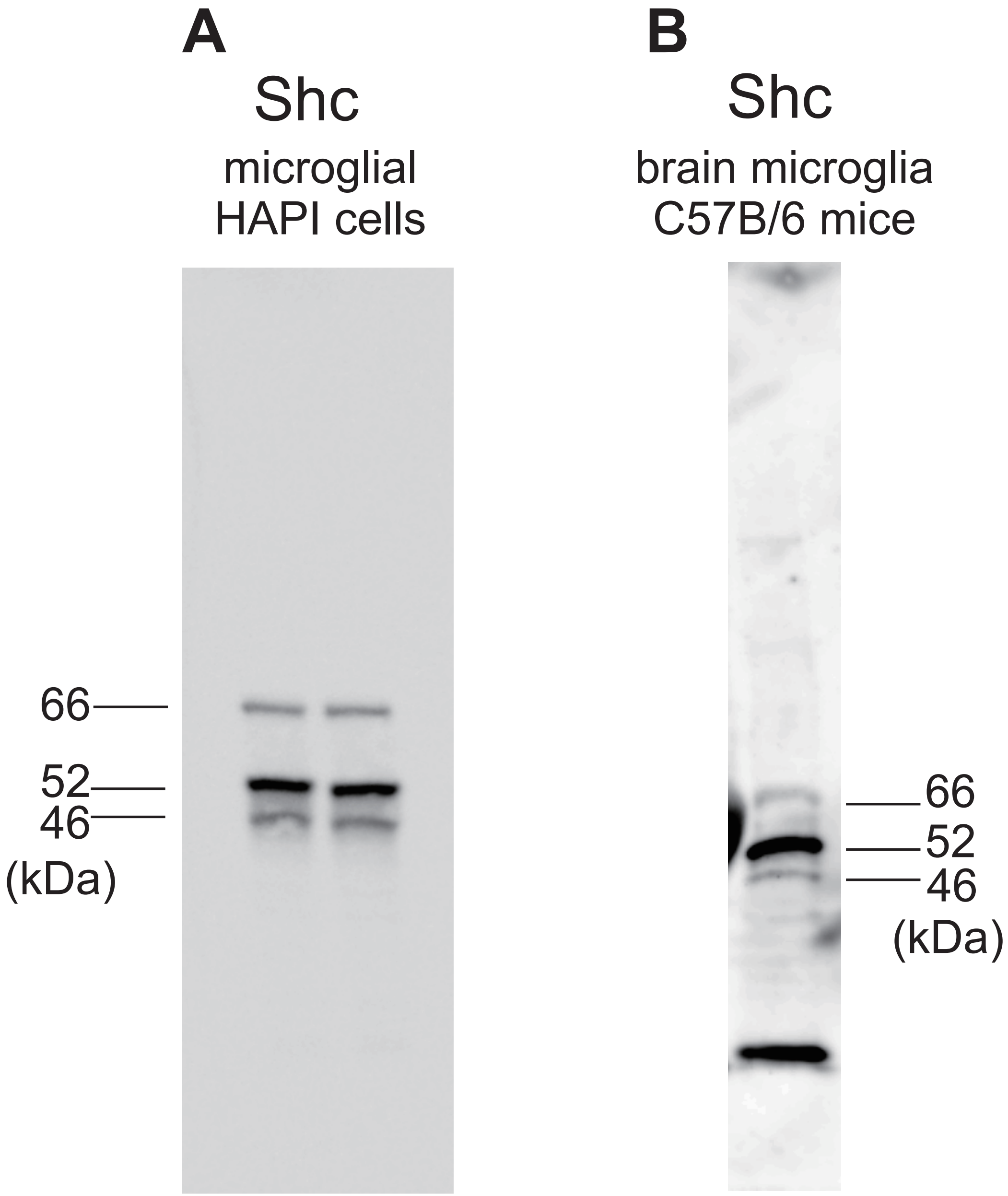

Figure S2: Western blot analysis of microglial cells.
Related to Figure 6.
Western blotting results using anti-Shc protein antibody, (A) with total protein extracted from microglial HAPI (highly aggressively proliferating immortalized) cells, and (B) with total protein extracted from brain microglia of C57B/6 mice.
